# ﻿Turbo taxonomy approaches: lessons from the past and recommendations for the future based on the experience with Braconidae (Hymenoptera) parasitoid wasps

**DOI:** 10.3897/zookeys.1087.76720

**Published:** 2022-02-25

**Authors:** Jose L. Fernandez-Triana

**Affiliations:** 1 Canadian National Collection of Insects, Ottawa, Canada Canadian National Collection of Insects Ottawa Canada

## Abstract

Not aplicable to a Forum paper, but if needed I can write one.

## ﻿Introduction

A recent paper (Sharkey et al. 2021a) describing 416 new species of Braconidae parasitoid wasps (Hymenoptera) from Area de Conservación Guanacaste in Costa Rica has reignited the debate about taxonomic best practices when describing species. The new species were treated in a minimalist way, as stated in the very title of the paper and this quote from their abstract: “Most descriptions consist of a lateral or dorsal image of the holotype, a diagnostic COI consensus barcode, the Barcode Index Number (BIN) code with a link to the Barcode of Life Database (BOLD), and the holotype specimen information required by the International Code of Zoological Nomenclature” (Sharkey et al. 2021a: 2).

Sharkey et al. (2021a) is but the latest example of a growing list of papers that aim to accelerate the description of species on a planet facing a biodiversity crisis in which species may become extinct before they are even described. “Turbo taxonomy” is a catchy name proposed by Butcher et al. (2012) to qualify such papers, and it has been rather enthusiastically applied since then – a Google Scholar search for “turbo taxonomy” retrieved 135 results since 2012 through January 2022. A similar concept “fast-track taxonomy” was proposed around the same time by Riedel et al. (2013a); I consider them as equivalent and for the sake of simplicity I will use “turbo taxonomy” henceforth.

Although somewhat subjective, turbo taxonomy can be characterized as the rapid description of many species in “fast” papers (as compared to the “slower” pace of traditionally produced taxonomic papers). This is usually accomplished using a combination of tools and approaches to automate and expedite dealing with the material examined, e.g., morphological traits quickly assessed and scored, often with brief descriptions and/or descriptions generated using software packages, high-quality illustrations, a heavy reliance on molecular and other data (e.g., biological, distributional) to differentiate and diagnose species. The combination of techniques for species recognition and description at least partially intersects with another concept, that of “integrative taxonomy”, sensu Dayrat (2005), and perhaps sometimes both terms have been used interchangeably, although integrative taxonomic papers are not necessarily “rapidly produced” as is claimed for the turbo-taxonomy ones.

The main difference between Sharkey et al. (2021a) relative to previous turbo-taxonomy papers, and the reason for the present discussions within the scientific community is that they chose to describe the new species based almost exclusively on DNA barcodes.

Describing new species based only or mostly on molecular data is not new. Hibbett et al. (2011) discussed prospects for sequence-based taxon discovery and description in fungi (see also Taylor 2011; Kõljalg et al. 2013); and Renner (2016) compiled a list of at least 98 names of species of acoels, lichens, angiosperms, annelids, alveolates, arachnids, centipedes, turtles, fishes, butterflies, mollusks, nematodes, and pathogenic fungi that have been published based on diagnostic mitochondrial, plastid, or nuclear DNA substitutions, indels, or rarely genetic distances, with or without the addition of morphological features. Even within Braconidae, some of the coauthors of Sharkey et al. (2021a) had recently published a similar, albeit much smaller paper (Meierotto et al. 2019).

Thus, the novelty of the Sharkey et al. paper is hardly the approach itself but rather the scaling up of the work to a mammoth monograph in which more than 400 new species were described. That is indeed a first. And, as quoted from the very first sentence of their introduction, the authors presented their article as a way to “further refine methods to overcome the taxonomic impediment of ichneumonoid biodiversity” (Sharkey et al. 2021a: 6).

In the months following that paper, the scientific community has engaged in lively discussions about “how useful” such descriptions are, whether they in fact impede the cataloguing of biodiversity, “how valid” (from the ICZN perspective) those species are, and general issues about the future of taxonomy, and the shortcomings of BINs and even BOLD (e.g., Ahrens et al. 2021; Engel et al. 2021; Meier et al. 2021).

In this Forum Paper I discuss some of the above issues, present alternative/complementary ideas from my perspective, and include a detailed proposal on how to approach turbo taxonomy in a hyperdiverse group such as braconid parasitoid wasps balancing rapid descriptions of species while also keeping a higher use value of the final product(s). I do not claim to have better or newer insights than others, and I certainly do not pretend to have any definitive answers, but perhaps my comments could be useful because a) I am a braconid researcher, like the main authors of the Sharkey et al. (2021a) paper, b) I have published several papers that could be considered as turbo taxonomy and have long been interested in ways to speed up species descriptions, c) I was actually one of the reviewers of Sharkey et al. (2021a) (and for full disclosure, I recommended its acceptance, although I also added many opinions on its taxonomic approach and how it could have been improved, with many of my suggestions being ignored by the authors in the final version), and d) perhaps more importantly, because I think that Sharkey et al. (2021a), even if arguably flawed, demonstrate opportunities that can and should be used by the taxonomic community to improve and speed up work in the future. In that sense, what follows below is less of another critical view of that paper and more of a complementary proposal to improve turbo taxonomic methods.

## ﻿“Talking the talk and walking the walk” of turbo taxonomy

There are many published papers that discuss the need to and possibilities of speeding up taxonomy by using newer technologies such as DNA barcoding. Unfortunately, most of those papers present somewhat general discussions or are intended just as a proof of concept, without actually applying it to describing new species. In many cases, DNA barcoding is presented as a useful and comparatively rapid tool to rapidly distinguish species, often revealing a much higher species diversity than previously thought based on morphological study and/or revealing complexes of cryptic species. However, usually things stop there, and the next step is not made, i.e., the new taxa are not described in those papers praising how much DNA barcoding brings to the taxonomist’s table. I would consider those papers examples of “talking the talk” but not necessarily “walking the walk” (in the sense presented here: https://knowyourphrase.com/talk-the-talk). It is important to stress that this statement does not apply to the four braconid experts and coauthors of the Sharkey et al. (2021a) paper (Michael Sharkey, Scott Shaw, Donald Quicke, and Kees van Achterberg) all of whom are world-renowned taxonomists. Altogether they have described more than three thousand new species in hundreds of published papers (e.g., see Yu et al. 2016), and their contributions to our knowledge of Braconidae and other Hymenoptera groups has been outstanding. They have certainly walked the walk!

But the truth is that comparatively few works could have the turbo taxonomy label applied to them. Examples include lichens (Lücking et al. 2017), annelids (Summers et al. 2014), dragonflies (Dijkstra et al. 2015), frogs (Rakotoariso et al. 2017), phorid flies (Hartop and Brown 2014; Hartop et al. 2015, 2016), histers beetles (Caterino and Tishechkin 2013), weevils (Riedel et al. 2013b, 2014; Riedel and Tänzler 2016; Riedel and Narakusumo 2019), and several papers on braconids (Table 1). There is no doubt that other papers than the ones I list can be found in the literature, but they still constitute a minority of published taxonomic revisions. [Srivathsan et al. (2019) could also be considered here, albeit only partially, because they discuss and present novel methodologies for rapid description of species (= turbo taxonomy) but only describe one new species as an example].

**Table 1.** Selection of published Braconidae papers (2005–2021) that could be considered as examples of turbo taxonomy. For the sets of data in columns 5–9, the use of “-” means such data was not present in the paper, “+” means that it was used but only in a very basic and limited way, and “++” means that it was fairly used. ACG = Area de Conservación de Guanacaste, Costa Rica.

Paper	Subfamily/ genus covered	Main geographical area	Total species /new species described	Use of dichotomous keys	Use of morphological data	Use of illustrations	Use of molecular data	Use of other data
Sharkey et al. (2021a)	11 Subfamilies of Braconidae	ACG	416/403	–	–	+	++	+
Marsh et al. (2013)	Doryctinae/ *Heterospilus*	Costa Rica	286/280	++	++	++	–	–
Fernandez-Triana et al. (2014)	Microgastrinae/ *Apanteles*	ACG	205/186	++	++	++	++	++
Butcher et a. (2012)	Rogadinae/ *Aleiodes*	Thailand	186/179	++	++	++	++	–
Arias-Penna et al. (2019)	Microgastrinae/ *Glyptapanteles*	ACG/ Ecuador	136/136	++	++	++	++	++
Liu et al. (2020)	Microgastrinae/*Apanteles*	China	97/48	++	++	++	–	+
Sharkey et al. (2018)	Agathidinae/ *Alabagrus*	ACG	87/66	++	++	++	++	++
Liu et al. (2019)	Microgastrinae/*Dolichogenidea*	China	67/39	++	++	++	–	+
Ahlstrom (2005)	Macrocentrinae/*Macrocentrus*	Nearctic	54/13	++	++	++	–	+
Valerio and Whitfield (2015)	Microgastrinae/ *Hypomicrogaster*	ACG	45/40	++	++	++	–	++
Fernandez-Triana et al. (2014)	Microgastrinae/*Pseudapanteles*	ACG	36/25	++	++	++	++	++
Liu et al. (2018)	Microgastrinae/*Dolichogenidea*	China	34/26	++	++	++	–	+
Fernandez-Triana et al. (2015)	Microgastrinae/ *Microplitis*, *Snellenius*	ACG	33/28	++	++	++	++	++
Meierotto et al. (2019)	Agathidinae/ *Zelomorpha*	ACG	19/18	–	–	+	++	++

What is somewhat surprising (or worrisome?) is the realization that few of the researchers who have published a paper that could be considered as turbo taxonomy have continued to do afterwards, i.e., they have not produced additional monographs in the same turbo taxonomy style. Based on my, admittedly non-exhaustive, online searches, I can only mention Riedel and colleagues for weevils (Riedel et al. 2013b, 2014; Riedel and Tänzler 2016; Riedel and Narakusumo 2019) and a series of papers on Braconidae (see Table 1 and discussion below) as two examples of researchers doing turbo taxonomy on a more sustained basis.

One may then ask, if turbo taxonomy is touted as “the way to move forward” in taxonomy, why are there so few adopters of the approach, and even fewer who repeat their efforts in subsequent papers? In my opinion the answer is simple: because turbo taxonomy still requires a significant amount of invested work and time, and it is not as easy and rapid as one might think or as it is purported to be in papers advocating for those revolutionary taxonomic approaches. A simple search of author names reveals that most of the published turbo taxonomy papers have been done primarily by graduate students (M.Sc. and Ph.D.) or postdoctoral fellows. They represent some of the more enthusiastic, hard-working, and “overperformer” researchers in the taxasphere, a great combination of youth, energy, and a desire/need to advance their careers. They certainly put in the effort needed to accomplish their turbo taxonomy feats, and they deserve all the praise for that. But could those papers become the “new normal” for taxonomy? I would argue that it is unrealistic to expect that turbo taxonomy papers can be produced effortlessly and quickly, much less in a sustained way, at least those closer to “traditional taxonomy” in the sense of providing keys and morphological descriptions.

I believe that Meierotto et al. (2019), Sharkey et al. (2021a), and others before them (see Introduction for non-Braconidae examples) are probably correct in their claim that a shift of paradigms is possible and needed to increase the speed of taxonomic results. I also agree that DNA-based species recognition should be one of the major driving forces to speed up the cataloguing of biodiversity. Where I disagree with such authors is in the way to implement turbo taxonomy because I believe that this can and should include components other than DNA that increase the “use value” of the paper while not taking much extra time or resources.

## ﻿Comparing the works of Meierotto and Sharkey with other Braconidae papers of similar size

First let us look at what has been accomplished with turbo taxonomy relative to Braconidae during the past 15 years or so (2005–present). Table 1 presents basic data on some papers, divided in two somewhat arbitrary categories. The first five rows include papers with the largest numbers of treated species (approximately 100–400 species each), to serve as a direct comparison with Sharkey et al. (2021a) which is, by far, the largest paper discussed here. Included are all the large monographs in Braconidae I am aware of that could be considered as examples of turbo taxonomy. The remaining rows contain a sample of papers with fewer treated species overall (approximately 30–80 each), which are comparable in size species-wise with Meierotto et al. (2019). There are certainly more examples of revisions of Braconidae in this second category than those I have listed.

Four of the large papers provide identification keys, “traditional” (i.e., morphology-based) species descriptions (as opposed to only DNA-based ones), and multiple illustrations of all or most species. The only exception to this is the paper of Sharkey et al. (2021a), which does not provide keys or traditional descriptions and includes only a single image per species (usually a lateral habitus). Molecular data to recognize, differentiate, and/or describe species was used in all papers except Marsh et al. (2013) and Liu et al. (2020). Other data, mostly biological information, usually host data but also number and shape of wasp cocoons, host plant, microhabitat, etc., were less prevalent, and mostly restricted to those papers treating the Area de Conservación de Guanacaste, Costa Rica (ACG) fauna because of the wealth of biological and ecological information available for Braconidae and other taxa obtained in that area (e.g., Janzen and Hallwachs 2011, 2016, 2020; see also http://janzen.sas.upenn.edu/caterpillars/database.lasso).

The pattern among the shorter papers is mostly similar, with Meierotto et al. (2019) being the only one not to include differential keys or morphological descriptions. All the other papers are more complete from the perspective of morphology, and many also included molecular, biological, and ecological data although, again, the ACG papers were more comprehensive because the authors had access to more information.

An interesting comparison can be drawn between the Meierotto et al. (2019) and Sharkey et al. (2018) papers: both treat a single genus of Agathidinae (Braconidae) but the latter is much more comprehensive in its use of features/traits to recognize, identify, and describe the species.

The examples in Table 1 are comprehensive taxonomic revisions that treated dozens and sometimes even hundreds of species each; they included at least some basic morphological data, usually more. Indeed, if a taxon could claim the crown of turbo taxonomy, Braconidae would be a strong candidate. In just one subfamily, Microgastrinae, a total of 720 new species was described between 2014 and 2019 (Fernandez-Triana et al. 2020), the vast majority in papers that would qualify as turbo taxonomy.

There is no question that these papers could have been produced faster and easier if a minimalistic approach, such as those of Meierotto et al. (2019) and Sharkey et al. (2021a), had been adopted. How fast and how easy are, however, complicated questions to answer. And how “useful” those papers would be for potential users is an even more difficult one.

## ﻿Speed, practicality, affordability, democratization of taxonomy, and Star Trek

Sharkey et al. (2021a), and for that matter many other papers, my own included, that have treated the ACG fauna benefited immensely from the work previously done by Daniel Janzen, Winnie Halwachs, and their team (e.g., Janzen et al. 2009; Janzen and Hallwachs 2011, 2016, 2020). Thanks to herculean efforts (including their amazing parataxonomists and technicians, mostly in Costa Rica but also in USA and Canada), thousands of specimens have been collected, reared, labelled, and databased with recorded host data, and DNA has been extracted, with the available sequences and additional information readily accessible in the Barcode of Life Data System (BOLD). Some of that work is highly technical, and all of it took a lot of time and significant resources, including financial. All or most of that was done before the actual work of the taxonomists started, and in fact was of critical importance or else it would have taken much more time and considerably more resources to produce those taxonomic papers, whether traditional or turbo taxonomy.

Thus, when considering papers that claim to be “fast” because they only rely on DNA-based descriptions, one must also consider hidden but significant amounts of work done prior to the taxonomy study. If time, expertise, and resources needed to obtain all the previous information on which the taxonomy is based were accounted for, then those papers would suddenly appear less quick and easy to produce than as advertised, at least relative to ACG studies.

Beyond time and resources not being properly assessed in a paper employing only DNA-based descriptions, there is a bigger issue. And that is the fact that any user of such a paper must, by default, obtain DNA data for their own specimens before any meaningful comparison can be made with the species dealt with in that paper. Otherwise, it is not possible to conclude if a specimen at hand belongs to a previously “DNA-described” species or is new. Thus, “DNA-only description” papers force users to do “DNA-only identifications”.

There is no problem with that, say some enthusiastic supporters of turbo taxonomy and DNA barcoding. It will actually democratize taxonomy because technical knowledge of a taxon, including the associated morphological jargon used to described it (e.g., number of setae on propodeum or sculpture on mesoscutum), would no longer be required. What used to be the domain of a relatively few taxonomists would become mostly unnecessary, because “soon” everyone would be able to use a device, à la Star Trek tricorder (https://en.wikipedia.org/wiki/Tricorder), to identify species. It would allow even school children to rapidly identify the caterpillar they found in their backyard or farmers in Central America to recognize which pest or parasitoid wasps are found in their crops. It all looks so nice and promising!

I fully agree that DNA barcoding democratizes taxonomy because indeed it reduces somehow the need for trained taxonomists to do routine identifications (e.g., Janzen et al. 2009; Janzen and Hallwachs 2001, 2016). But, while I have no doubts that technology ultimately will be developed to allow fast, easy, and cheap devices to obtain and analyse DNA, and access the comprehensive DNA databases that are necessary to determine whether a specimen at hand represents a described species, that scenario is not yet here (but see Srivathsan et al. 2021 for some new developments that could become viable alternatives in the near future). We are still far from being able to download a “Taxonomy for Dummies” app.

Meanwhile, what we have is the fact that DNA-based taxonomy is not accessible or affordable to everyone (see further analyses and/or other perspectives in Pinheiro et al. 2019; Dupérré 2020; Ahrens et al. 2021; Meier et al. 2021; Srivathsan et al. 2021). At present, it is not possible to obtain a DNA barcode from a single specimen unless the individual has access to a molecular lab, whether this is their own or “one for hire”. As an example of the latter, one of the most commonly used such labs is the Canadian Center for DNA Barcoding (formerly the Biodiversity Institute of Ontario), which charges $1,250 Canadian dollars for a single plate of 95 specimens (http://ccdb.ca/pricing/). However, in addition to that cost, single images of every submitted specimen and an Excel file with some basic information are also required when samples are submitted, which will take additional time and money; factors also to consider are the shipping costs and dealing with national/international laws regulating access, sharing, and exportation of genetic resources.

Never mind the school children or farmers, arguably most world researchers cannot afford the current costs and associated logistic challenges mentioned above to obtain DNA-based identifications for every specimen they may need or want to identify (e.g., Srivathsan et al. 2021). If the route of having to obtain DNA barcodes (or any other molecular marker) to identify species becomes the only route to a scientific name, then this could make taxonomy even less accessible and democratic than using “traditional” techniques such as microscopes and dichotomous keys. At present is certainly valid to argue that the cost of traditional, morphology-based taxonomy is largely a “front end” cost mainly borne by the taxonomist, whereas DNA-only taxonomy necessitates high and significant “back end” user costs.

In addition to cost and who pays this, there is also the problem of the almost two million species described in the pre-molecular era, many with no DNA associated. Those species cannot simply be ignored, as it has been claimed to be the case in the Meierotto et al. (2019) paper. Zamani et al. (2020) thoroughly discussed that problem, although Sharkey et al. (2021a, b) gave some counter replies.

In the end, it comes down to the practicality and benefits/damages that minimalistic (extreme?) taxonomic approaches, such as those relying only on DNA barcodes for species description and recognition, bring. Do future revisions to be produced really need to ignore morphology and previously described species to instead rely entirely or almost exclusively on DNA barcodes, with the “justification” of describing species faster because of the biodiversity crisis? Or is it possible to build upon the works of Meierotto et al. (2019), Sharkey et al. (2021a), and others to try finding a middle-of-the-road approach, where speed and practicality are attained while significantly minimizing efforts and cost?

## ﻿A “cookbook recipe” for turbo taxonomy, including estimated times needed for each task

What I propose below is a workflow and guidelines for preparing turbo taxonomy papers, including estimated times for each task. The main motivation is to provide an alternative to Meierotto et al. (2019) and Sharkey et al. (2021a) but with the addition of some features that I hope would increase the applicability of the work (from a user perspective) while still maintaining a relatively fast pace. I have based this proposal on my personal experience preparing Braconidae turbo taxonomy papers, but it could be adapted for other taxa, i.e., used like a “cookbook recipe” that can be modified and changed as needed or desired.

I do not pretend to reinvent the wheel, e.g., see Reidel et al. (2013), Hartop and Brown (2014), Srivathsan et al. (2019) for earlier turbo taxonomy proposals and even nicer workflow diagrams (although my proposal includes more detailed analyses of time involved with each task and consideration of other factors). I also strongly recommend checking the new guidelines for species descriptions posted by ZooKeys: https://zookeys.pensoft.net/about#TaxonomicTreatments), which in some ways intersects what I write below. And it may also be fruitful to check the many exchanged messages in the email list for biological systematics Taxacom (http://mailman.nhm.ku.edu/cgi-bin/mailman/listinfo/taxacom), where the Meierotto and Sharkey papers were vigorously discussed in 2019 and 2021 (while I have refrained from commenting on Taxacom about Sharkey et al. (2021a), in 2019 I did share my opinion about Meierotto et al. (2019), and some of the ideas presented here are based on what I wrote to that list at that time).

### ﻿a) When is it most efficient to use turbo taxonomy approaches?

- The taxon being studied is hyperdiverse, i.e., species-rich, and mostly poorly known, i.e., most species are still undescribed so there are relatively few names not previously associated with DNA data and type material to be considered.
- DNA barcodes are already available for many/most of the species, unless the research project has sufficient resources (time and money) to accomplish this step.
- Databasing of many/most specimens is already available, unless the research project has sufficient resource (time and money) to accomplish this step.
- Imaging equipment is available capable of generating many images in a short period of time and with automated or semi-automated capabilities of stacking images to produce publication-quality images.
- Other sources of data (biological, ecological, etc.) are available for many/most specimens that provide evidence of species status supplemental to DNA/morphology evidence.
- A ‘minimum’ set of morphological traits to assess specimens is already available, i.e., features have been discussed or proposed in previous studies of the taxon or related taxa by specialist(s) in the taxon in order to provide supplementary evidence of species status and which is necessary for more “traditional” taxonomic approaches. Alternatively, the paper to be produced presents such a set of minimum morphological traits.

### ﻿b) Species treatment

- New species will be treated, diagnosed, and described using a combination of basic morphology (basic key and brief diagnostic description), molecular data when available (e.g., DNA barcodes), ecological/ethological data when available, distribution data, and complete details of the primary type(s) and basic details of all other specimens.
- Previously described species will be incorporated into the paper even if in an incomplete manner due to lack of molecular or other data.

### ﻿c) Use of morphological data

Simplified key(s) and diagnostic descriptions, with a minimum set of morphological traits, will be prepared. The morphological traits, ideally chosen by a specialist in the taxon, need not be numerous but ideally should be easily and quickly assessed and scored (i.e., not requiring dissections, slide preparation, or other labour-intensive techniques). It is understood that DNA evidence likely is being used in most turbo taxonomy studies because of a perceived lack of differential morphological features for the group, and that morphology will not necessarily suffice to tell every species apart. However, morphology should at least be able to place most (ideally all) species within some sort of smaller group of species. A “species group”, as here considered, is based on some simple, diagnosable trait(s), e.g., “all species with legs brown or black versus all species with legs yellow” and does not necessarily have to be monophyletic.

The morphology component of the taxonomic revision should serve as the minimum piece of information to allow someone with a basic knowledge of the taxonomic group and simple equipment such as a microscope to recognize a species or species group if no other source of information, such as DNA, is available. [This statement may not be applicable in some groups, such as nematodes, fungi, etc. The present paper was mostly written thinking of insects, and it is mainly directed to groups where morphology has some role in recognizing species or groups.]

Although diagnostic descriptions should be as short as possible based on easily observable features, each species should be illustrated as fully as possible with images showing body areas from different angles in order to document the features important for differentiating species in the group (e.g., coloration, sculpture, etc.) and those features that are otherwise not described. Ideally, illustrations should be based on the holotype or specimens compared with the holotype; if a species is thought to be variable morphologically, then specimens showing the perceived range in variation should also be photographed.

In species complexes with very similar or cryptic morphology, additional effort does not necessarily need to be spent trying to separate them based on detailed study of morphology or morphometrics, but instead other non-morphological criteria (see below), if known, could be used to help distinguish the species.

The estimated time needed for the morphological work is 5 hours per species. This includes scoring and writing the species description based on minimum morphological traits, and also includes studying intraspecific variation and making a few measurements of relevant structures. All of these steps should take, on average, less than one hour per species, the exception being species with many available specimens and/or significant morphological variation. To account for extremes, an estimate of two hours of work per species is considered here. Photographing a species (4–8 shots of a specimen, to capture different angles) can be done in one hour depending on the number of specimens per species imaged, and the photographic equipment and montaging software used. Preparing a plate of images can be done in less than one hour. Estimating the time to prepare a simplified key is very difficult, and here a conservative estimate of one hour per species in the key is proposed. [Obviously, the calculations for this point do not include the years of taxonomic experience that are required to be able to describe a species in 5 hours. This is indeed another “hidden prior work” and time to factor in. However, it would not only apply similarly to both turbo taxonomy and any other taxonomic approaches but also it would be very difficult, if not impossible, to calculate; thus, that factor is not included here. One simple observation from that problem would be that we still need to have more trained taxonomists to do the work of describing new species!].

### ﻿d) Use of molecular data

DNA barcoding and/or any other molecular marker will be a very important criterion to recognize and diagnose species, and for morphologically cryptic or very similar species, it may be the primary criterion. Species will be characterized as much as possible by their corresponding Barcode Index Number (BIN) (for a definition of BIN see Ratnasingham and Hebert 2013). If a unique BIN does not “work”, i.e., in cases where there is more than one BIN per species or several species share the same BIN, a discussion explaining the rationale to characterize the species molecularly will be necessary.

Where a species is primarily defined and identified by DNA barcodes because, e.g., basic morphology is insufficient or inconclusive, such “DNA-only species” must include sequences from at least two different specimens (to exclude potential definition of a species based on a single sequence, which could be a lab contamination, a chimera, or any other error). Where a species is defined by a combination of traits (morphological, biological, etc.), a less stringent molecular criterion is acceptable, and a single DNA barcode can be sufficient.

The estimated time needed for the molecular tasks is 5 hours per species. Sampling tissue for DNA barcoding from dry, pinned specimens is straightforward and takes less than 10 minutes per specimen. However, the associate requirements for preparing a 96-well plate and submitting it to the lab for processing may require many other tasks, e.g., taking one image per specimen and providing some details of the specimen for the BOLD database (in the case where specimen tissue is sequenced by the Canadian Center for DNA barcoding). A conservative estimate of 30 minutes per specimen is proposed. Because, as discussed above, it is usually necessary to have DNA barcodes of more than one specimen per species, the estimated here includes 3 hours per species. This estimate will vary significantly if specimens are prepared in batches smaller or larger than one 96-wells plate (which accommodates 95 specimens). Basic analysis of DNA barcodes (Neighbour-Joining trees as generated in BOLD) can be done quickly, but more complex and comprehensive analyses will take longer; a conservative estimate of 2 hours per species is proposed here.

### ﻿e) Use of ecological/ethological data

Any extra information that contributes to recognizing or identifying a species based on ecological or ethological traits should be used as additional evidence supporting species delimitation, but not as the single source to describe a species. Examples in Braconidae include host data, parasitoid ecology, wasp seasonality, etc.

The estimated time needed for the ecological/ethological tasks is 1 hour per species, though this greatly depends on the available information for each taxon; it could be significantly less or even zero. This and the following are probably the least accurate time estimates of the list.

### ﻿f) Use of distribution data

The minimum standard should be broad geographical distribution, i.e., biogeographical region, country, although detailed locality data is preferable. Information on habitat, e.g., collected in a rainforest or finer details, e.g., collected on understory of forest, on leaves of plant X, should also be provided when available. Distribution data can be used as supplementary evidence supporting a species delimitation and/or recognition, but not as the single source to describe a species.

The estimated time needed for the distribution data task is 1 hour per species, depending on the number of specimens to be data-mined and their geographic breadth, i.e., the amount of data available, and how much of that information is already databased.

### ﻿g) Dealing with primary type(s) and other specimens

Details of the name-bearing specimens (primary types) should be provided that minimally meet International Code of Zoological Nomenclature (ICZN) publication requirements, such as type depository, but also including the specimen’s unique identifier, specimen sex, country and other information on type specimen label(s) (photographs of such labels can be included), and any other detail (e.g., “specimen in good condition” or “missing a leg”) that facilitates the unambiguous recognition of the name-bearing type(s). The ZooKeys guidelines mentioned above are a great standard to follow.

For paratypes and other non-type specimens, considerably abbreviated data can be included. For example, just mentioning the unique identifiers for each specimen instead of detailing all the data for every specimen data is sufficient, as long as the unique identifiers are linked to a publicly available database or dataset where more detailed information is available.

The estimated time needed for dealing with specimen details is 1 hour per species, depending on the number of specimens and prior databasing. If most specimens are already databased, as is becoming more the norm in many collections, then the time may be less than 10 minutes for every primary type and another 10 minutes to record the unique identifiers of all other specimens.

### ﻿h) Treating previously described species

Previously described species should not be ignored, i.e., all species treated in a new paper should not, by default, be considered as new species if there are prior available names. Instead, effort should be made to incorporate the previously described species including a reasonable effort to locate and study their types and/or authenticated material. Admittedly, there will be instances when this is not possible and the only data available is just a prior, possibly uninformative, and very short description. However, even if only incomplete information is available for previously described species this should be discussed in the paper as far as possible. Two hypothetical examples are discussed below.

The most extreme example would be that of a previously described species known only from the missing holotype, already lost, and a useless original description a few words long. Such a species should still be dealt with in a manner like this: “Species A cannot be run though our key because it is impossible to assess morphological traits X, Y, and Z used in the key and the only known specimen is lost. Thus, it is not possible to determine whether the name applies to one of the new species described here, but for practical purposes we assume that is not the case.” Statements like that would make clear to the user/reader that such names cannot be presently assigned, and may never be, while still allowing progress in describing any new species.

Most cases will be less extreme than the above, with most previously described species being able to be placed within some context of the taxonomic revision, i.e., compared with the new species being described. Included should be at least some sort of basic statement such as: “Species B can only be run to couplet 3 of our key, as characters X and Y (from our key) cannot be assessed for that species, and therefore the name could potentially apply to species C, D, or E (new species being described in our paper), but for practical purposes we assume it is none of them”. Again, this method reduces the potential number of names that could (eventually) be found to be synonyms (as at least the species keyed out through the first two couplets would not), while still enabling the new, better characterized species to be recognized.

In these two hypothetical cases, the previously described species are not ignored, even if their status can never be properly assessed. Thus, the new taxonomic revision would bring together all available information, including presenting the shortcomings and gaps in our current knowledge of some species.

The estimated time needed for dealing with previously described species is, conservatively, 2 hours per species, though it will depend on all factors discussed above.

### ﻿i) Overall estimate time to deal with one species

The sum of all the time estimates above renders a total of 15 hours per species. That is roughly two days of work per species, or 2.5 species per week. Rounding down to 2 species per week and 50 weeks per year, one arrives at an estimate of 100 new species described in one full-time year of work by a turbo taxonomy practitioner.

However, how accurate is this estimate? Are there examples of this in the real world, or is the above just a theoretical, futile exercise?

It is difficult to get actual data from previous turbo taxonomy papers as to the time it took to complete the work because this is rarely (or never) stated by the author(s). But some information is available and other can be guessed.

I have no exact knowledge of how much time it took Sharkey et al. (2021a) to prepare their paper, but from correspondence with some of the coauthors I know that it took at least two years. Assuming that was the case (and not longer), it would mean a rate of 200 new species per year, an impressive number. But one needs to factor in how much time was spent by the other three coauthors of that paper who are braconid taxonomists, in addition to the primary author. As such, I suspect that the actual number is below 200 species described per year.

Many of the other larger papers listed in Table 1 represent the work of a Ph.D. thesis or postdoctoral research, each of which probably included at least 3 years of work with the specimens. Based on the total number of species for those revisions, that would give values between 40 and 100 species per year per paper.

Fortunately, I can provide a more accurate estimate for my own work revising *Apanteles* (Braconidae) in Mesoamerica (Fernandez-Triana et al. 2014), which took two years to complete. The revision treated 205 species and at the time I was working full time on the project. Consequently, the pace was approximately 100 species per year. But, very importantly, I benefited greatly from previous work accomplished by Dan Janzen and Winnie Hallwachs in ACG, and some preliminary sorting of species by James Whitfield (University of Illinois at Urbana-Champaign) and his students before I started – all those contributors were rightfully included as coauthors. Thus, the pace to produce that paper is not as fast as it would first appear, and it underscores the difficulties in calculating the actual amount of time it takes to produce comprehensive taxonomic revisions. If anything, I cannot take much credit for the results of that paper (more criticism of my own work below).

Another factor to consider is that a rate of 100 species/year can only be accomplished if treating species “in bulk”, i.e., if the purported review would include many new species. But not all taxonomic groups to be studied have hundreds of undescribed species and a taxonomic revision of “just” a dozen species would not be as time efficient. Furthermore, most people cannot spend 100% of their time doing taxonomic revisions. Even Ph.D. students have other things to do than just taxonomic revisions! Thus, a rate of 100 species/year is, in my opinion, a very high and somewhat unfair standard to expect, much less to meet on a consistent, year to year, basis; at least with current technology.

However, regardless of the actual time used for any taxonomic revision, efficiencies can be realized, such as including brief descriptions instead of traditional, longer, and more comprehensive ones, as proposed above. Going back to the real-world example of my own *Apanteles* paper, for that work I measured and scored 49 morphological characters (altogether more than 15,000 measurements). Many of those characters ultimately proved to be uninformative to distinguish species, being repetitive, too variable, or too subjective or complex to assess. In retrospect, the keys were also unnecessarily long, and some species almost impossible to tell apart based on the keys only (Eduardo Shimbori, pers. comm.). Looking back, eight years after I completed that paper in 2013, I see many inefficiencies in my work, and much superfluous data that could have been eliminated. Had I chosen a lower number of morphological characters and simplified the keys, it could have been completed quicker, without diminishing the final quality of the work. Had I assumed an approach similar to my proposed “cookbook recipe” above, the species would have been mostly recognized by DNA and host data, and the keys would have been constructed to serve a more basic and limited function than what I had intended, while still retaining some utility to recognize basic species-groups. Of course, one could argue that the potential value of any character cannot be comprehended until it has been analyzed. One cannot know that there are “x” number of useful characters, and what they are, prior to studying them. This is what research is all about. Perhaps the “useless” time spent on some measurements is actually an example of what is necessary and a part of all taxonomic revisions, unless morphological features are completely ignored.

**Figure 1.**
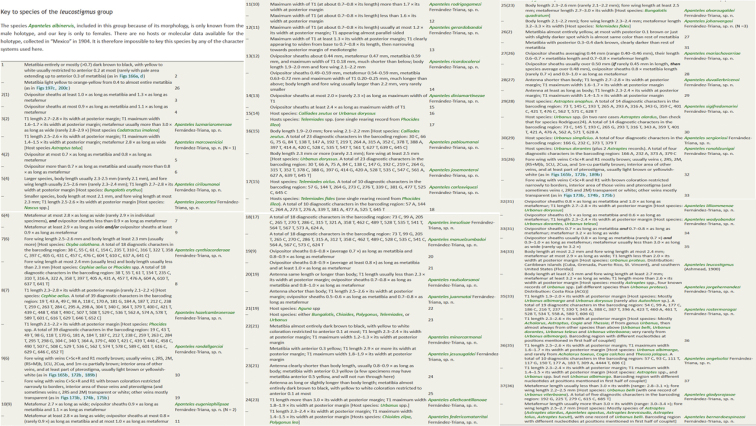
Details of the key to the *Apantelesleucostigmus* species group as it appeared in Fernandez-Triana et al. (2014). The plate shows a composite image of the key in the same format it appeared in the online version of that key (https://zookeys.pensoft.net/articles.php?id=3394).

**Figure 2.**
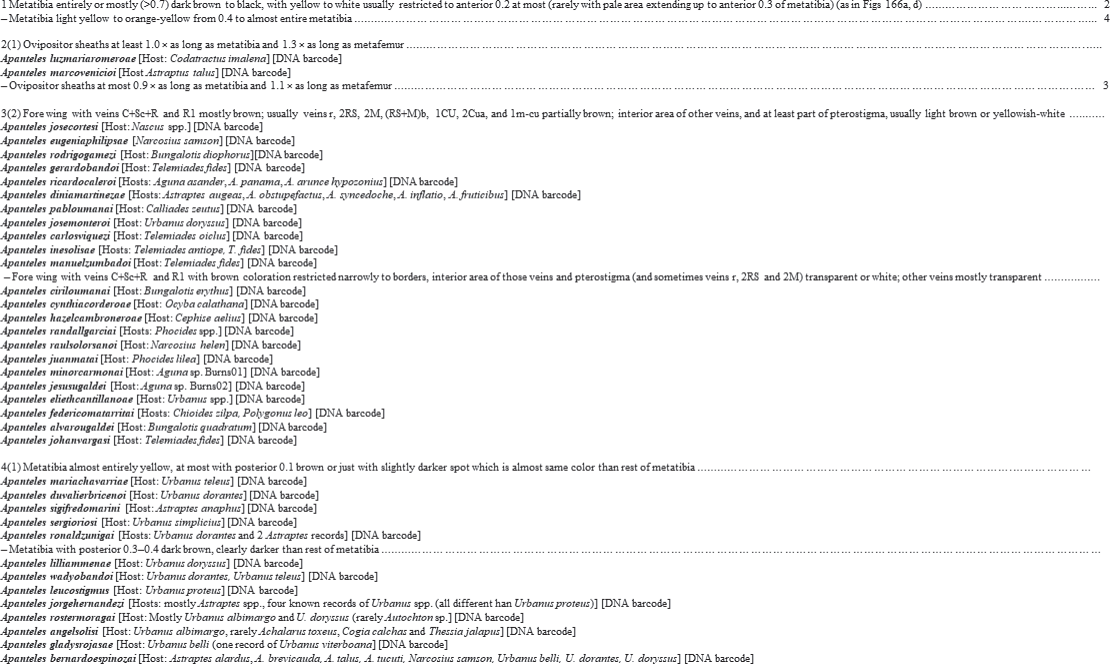
Details of the key to the *Apantelesleucostigmus* species group as it would look based on modifications detailed in the present paper (see section “h) Overall estimated time to deal with one species” in the current manuscript).

One example of how work can be reduced and made faster but still retain value is the case of the *Apantelesleucostigmus* species group, which comprises 39 species and is, by far, the largest and most difficult group of *Apanteles* to recognize and separate species in Mesoamerica. The key from Fernandez-Triana et al. (2014) for that group (reproduced here in Fig. 1) starts by dealing with a species that cannot be keyed out due to lack of data, with only one specimen known, and is an actual example on how to deal with historical species where information is not available. The remaining 38 species are keyed out using some characters difficult to assess and at some points the differences between halves of the same couplet are very subtle (the paper also included 4–8 images each of the adult wasps for every species). This key may look good on paper, but in practice it is very difficult and prone to error. Indeed, morphology does not work well for this group, which is suspected to include several morphologically cryptic species. Instead, I could have prepared a much simpler key that only used a few characters that are relatively easy to assess. Obviously, some species would end in the same point of the key, and thus could only be reliably identified by molecular and biological data. Such a “new” key (Fig. 2) would be much shorter and thus faster to prepare. As for the user of such key, there would still be the need of obtaining DNA barcodes and/or host data to obtain species identifications, but even if the user does not have such data, specimens could still be placed at least in some sub-group.

The above example, which I chose because it was the most difficult and problematic group of the *Apanteles* revision, illustrates how a mostly-but-not-only DNA based paper could be constructed in a more time-effective way. Other *Apanteles* groups from that Fernandez-Triana et al. (2014) revision (and indeed many groups in other taxa) might work even better. The proposed methods could shorten the time to produce a taxonomic revision while still providing some basic elements of more traditional papers.

## ﻿Concluding remarks

I do not pretend that my suggestions above will “solve” the problem of describing millions of additional species in a short period of time. Even a “fast” pace of 100 species/year per taxonomist would still take a few hundred years to finish the task, a luxury we cannot afford, or would require a significant increase in the number of professional taxonomists (an unlikely scenario). There is no easy or simple answer to the necessity (and urgency!) of accelerating taxonomic inventories. My opinion is that it will require a wide embracement of current and additional technology advances, but also some consensus-building among the taxonomic community on how to move forward, and perhaps even a broader involvement of citizen science. The present paper must be seen only as a modest attempt to provide some alternatives, even if insufficient. For some different perspectives and opinions on these topics, I recommend the reading of what the reviewers of the present paper had to say (Suppl. material 1).

It is very telling to see how many strong reactions a single paper has awakened in just a few months after its publication (or two papers, if we account for Meierotto et al. 2019), and the reasoning and pleas of other colleagues to avoid a future à la Sharkey et al. (2021a). I strongly recommend the reading of papers such as Pinheiro et al. (2019), Dupérré (2020), Zamani et al. (2020), Ahrens et al. (2021), Engel et al. (2021), Meier et al. (2021), Srivathsan et al. (2021), and references cited therein (other papers providing slightly different alternatives or approaches are also recommended reading, e.g., Brower (2010), Blaxter (2016), Goulding and Dayrat (2016), Renner (2016), Brown and Wong (2020), Vences (2020); this list is not exhaustive). And to present a more complete and fairer picture, the reader should also consider a second paper by Sharkey et al. (2021b) which tried to provide counterarguments to some of the received criticism, although that paper has also been met with additional counterarguments on its own, e.g., Ahrens et al. (2021), Engel et al. (2021), and Meier et al. (2021).

The authors cited in the previous paragraph have discussed in a more coherent, compelling, and convincing way that I probably could about the dangers and shortcomings of approaches such as those of Meierotto et al. (2019) and Sharkey et al. (2021a). While I agree with most of those arguments, I also think that the Meierotto and Sharkey papers provide an opportunity to critically look at and improve our own work. In that sense I prefer to be optimistic and focus on examples and the potential of what could be done (or has already been done by other authors) so that future turbo taxonomy papers can accomplish the (very much needed) dual goal of being fast and useful for the scientific community and the general public.

## Supplementary materials

Supplementary material 1 Comments from the reviewers Data type docx file Explanation note Comments from the reviewers of the manuscript, ordered as they were submitted. All reviewers agreed to be named in this supplementary file. https://binary.pensoft.net/file/651034This dataset is made available under the Open Database License (http://opendatacommons.org/licenses/odbl/1.0/). The Open Database License (ODbL) is a license agreement intended to allow users to freely share, modify, and use this Dataset while maintaining this same freedom for others, provided that the original source and author(s) are credited.Jose L. Fernandez-Triana

## References

[B1] Ahlstrom KR (2005) Revision of the subfamily Macrocentrinae (Hymenoptera: Braconidae) in America north of Mexico. Thomas Say Publications in Entomology: Monographs. Entomological Society of America, 274 pp.

[B2] Ahrens D Ahyong ST Ballerio A Barclay MV Eberle J Espeland M Huber BA Mengual X Pacheco TL Peters RS Rulik B Vaz-deMello F Wesener T Krell FT (2021) Is it time to describe new species without diagnoses?–A comment on Sharkey et al. (2021). Zootaxa 5027(2): 151–159. 10.11646/zootaxa.5027.2.1 34811237

[B3] Arias-Penna DC Whitfield JB Janzen DH Hallwachs W Dyer LA Smith MA Hebert PDN Fernández-Triana JL (2019) A species-level taxonomic review and host associations of *Glyptapanteles* (Hymenoptera, Braconidae, Microgastrinae) with an emphasis on 136 new reared species from Costa Rica and Ecuador. ZooKeys 890: 1–685. 10.3897/zookeys.890.35786 31798309 PMC6881475

[B4] Blaxter M (2016) Imagining Sisyphus happy: DNA barcoding and the unnamed majority. Philosophical Transactions of the Royal Society B: Biological Sciences 371: e20150329. 10.1098/rstb.2015.0329 PMC4971181 27481781

[B5] Brower AVZ (2010) Alleviating the taxonomic impediment of DNA barcoding and setting a bad precedent: names for ten species of ‘*Astraptesfulgerator*’ (Lepidoptera: Hesperiidae: Eudaminae) with DNA-based diagnoses, Systematics and Biodiversity 8(4): 485–491. 10.1080/14772000.2010.534512

[B6] Brown BV Wong MA (2020) Identification of *Megaselia* (Diptera: Phoridae) species using wing vein landmarking, Journal of Natural History 54: 37–38, 2513–2527. 10.1080/00222933.2020.1856431

[B7] Butcher BA Smith MA Sharkey MJ Quicke DL (2012) A turbo-taxonomic study of Thai *Aleiodes* (Aleiodes) and Aleiodes (Arcaleiodes) (Hymenoptera: Braconidae: Rogadinae) based largely on COI barcoded specimens, with rapid descriptions of 179 new species. Zootaxa 3457(1): 1–232. 10.11646/zootaxa.3457.1.1

[B8] Caterino M Tishechkin A (2013) A systematic revision of *Operclipygus* Marseul (Coleoptera, Histeridae, Exosternini). ZooKeys 271: 1–401. 10.3897/zookeys.271.4062 PMC3652427 23717185

[B9] Dayrat B (2005) Towards integrative taxonomy. Biological Journal of the Linnean Society 85(3): 407–417. 10.1111/j.1095-8312.2005.00503.x

[B10] Dijkstra KDB Kipping J Mézière N (2015) Sixty new dragonfly and damselfly species from Africa (Odonata). Odonatologica 44(4): 447–678.

[B11] Dupérré N (2020) Old and new challenges in taxonomy: what are taxonomists up against? Megataxa 1(1): 59–62. 10.11646/megataxa.1.1.12

[B12] Engel MS Ceríaco LMP Daniel GM Dellapé PM Löbl I Marinov M Reis RE Young MT Dubois A Agarwal I Lehmann P Alvarado M Alvarez N Andreone F Araujo-Vieira K Ascher JS Baêta D Baldo D Bandeira SA Barden P Barrasso DA Bendifallah L Bockmann FA Böhme W Borkent A Brandão CRF Busack SD Bybee SM Channing A Chatzimanolis S Christenhusz MJM Crisci JV D’elía G Da Costa LM Davis SR De Lucena CAS Deuve T Fernandes SE Faivovich J Farooq H Ferguson AW Gippoliti S Gonçalves FMP Gonzalez VH Greenbaum E Hinojosa-Díaz IA Ineich I Jiang J Kahono S Kury AB Lucinda PHF Lynch JD Malécot V Marques MP Marris JWM Mckellar RC Mendes LF Nihei SS Nishikawa K Ohler A Orrico VGD Ota H Paiva J Parrinha D Pauwels OSG Pereyra MO Pestana LB Pinheiro PDP Prendini L Prokop J Rasmussen C Rödel MO Rodrigues MT Rodríguez SM Salatnaya H Sampaio I Sánchez-García A Shebl MA Santos BS Solórzano-Kraemer MM Sousa ACA Stoev P Teta P Trape JF Van-Dúnem Dos Santos C Vasudevan K Vink CJ Vogel G Wagner P Wappler T Ware JL Wedmann S Zacharie CK (2021) The taxonomic impediment: a shortage of taxonomists, not the lack of technical approaches. Zoological Journal of the Linnean Society 193(2): 381–387. 10.1093/zoolinnean/zlab072

[B13] Fernandez-Triana J Whitfield J Rodriguez J Smith M Janzen D Hajibabaei M Burns J Solis A Brown J Cardinal S Goulet H Hebert P (2014) Review of *Apanteles* sensu stricto (Hymenoptera, Braconidae, Microgastrinae) from Area de Conservación Guanacaste, northwestern Costa Rica, with keys to all described species from Mesoamerica. ZooKeys 383: 1–565. 10.3897/zookeys.383.6418 PMC3950464 24624021

[B14] Fernandez-Triana J Janzen D Hallwachs W Whitfield J Smith M Kula R (2014) Revision of the genus *Pseudapanteles* (Hymenoptera, Braconidae, Microgastrinae), with emphasis on the species in Area de Conservación Guanacaste, northwestern Costa Rica. ZooKeys 446: 1–82. 10.3897/zookeys.446.8195 PMC4205727 25349512

[B15] Fernandez-Triana JL Whitfield JB Smith MA Kula RR Hallwachs W Janzen DH (2015) Revision of the genera *Microplitis* and *Snellenius* (Hymenoptera, Braconidae, Microgastrinae) from Area de Conservación Guanacaste, Costa Rica, with a key to all species previously described from Mesoamerica. Deutsche Entomologische Zeitschrift 62(2): 137–201. 10.3897/dez.62.5276

[B16] Fernandez-Triana J Shaw MR Boudreault C Beaudin M Broad GR (2020) Annotated and illustrated world checklist of Microgastrinae parasitoid wasps (Hymenoptera, Braconidae). ZooKeys 920: 1–1089. 10.3897/zookeys.920.39128 32390740 PMC7197271

[B17] Goulding TC Dayrat B (2016) Integrative taxonomy: ten years of practice and looking into the future. In: Аспекты биоразнообразия (Biodiversity Aspects). Archives of Zoological Museum of Lomonosov Moscow State University, 116–133.

[B18] Hartop E Brown B (2014) The tip of the iceberg: a distinctive new spotted-wing *Megaselia* species (Diptera: Phoridae) from a tropical cloud forest survey and a new, streamlined method for *Megaselia* descriptions. Biodiversity Data Journal 2: e4093. 10.3897/BDJ.2.e4093 PMC4238073 25425935

[B19] Hartop EA Brown BV Disney RHL (2015) Opportunity in our ignorance: urban biodiversity study reveals 30 new species and one new Nearctic record for *Megaselia* (Diptera: Phoridae) in Los Angeles (California, USA). Zootaxa 3941(4): 451–484. 10.11646/zootaxa.3941.4.1 25947525

[B20] Hartop E Brown B Disney R (2016) Flies from L.A., The Sequel: A further twelve new species of *Megaselia* (Diptera: Phoridae) from the BioSCAN Project in Los Angeles (California, USA). Biodiversity Data Journal 4: e7756. 10.3897/BDJ.4.e7756 PMC4867692 27226746

[B21] Hibbett DS Ohman A Glotzer D Nuhn M Kirk P Nilsson RH (2011) Progress in molecular and morphological taxon discovery in Fungi and options for formal classification of environmental sequences. Fungal Biology Reviews 25(1): 38–47. 10.1016/j.fbr.2011.01.001

[B22] Janzen DH Hallwachs W (2011) Joining inventory by parataxonomists with DNA barcoding of a large complex tropical conserved wildland in northwestern Costa Rica. PLoS ONE 6(8): e18123. 10.1371/journal.pone.0018123 PMC3156711 21857894

[B23] Janzen DH Hallwachs W (2016) DNA barcoding the Lepidoptera inventory of a large complex tropical conserved wildland, Area de Conservacion Guanacaste, northwestern Costa Rica. Genome 59(9): 641–660. 10.1139/gen-2016-0005 27584861

[B24] Janzen DH Hallwachs W (2020) Área de Conservación Guanacaste, northwestern Costa Rica: Converting a tropical national park to conservation via biodevelopment. Biotropica 00: 1–20. 10.1111/btp.12755

[B25] Janzen DH Hallwachs W Blandin P Burns JM Cadiou J Chacon IS Dapkey T Deans AR Epstein ME Espinoza B Franclemont JG Haber WA Ibabaei MH Hall JP Hebert PDN Gauld ID Harvey DJ Hausmann A Kitching IJ Lafontaine D Landry J-F Lemaire C Miller JE Miller JS Miller L Miller SE Montero J Munroe E Green SR Ratnasingham S Rawlins JE Robbins RK Rodriguez JJ Rougerie R Sharkey MJ Smith MA Solis MA Sullivan JB Thiaucourt P Wahl DB Weller SJ Whitfield JB Willmott KR Wood DM Woodley NE Wilson JJ (2009) Integration of DNA barcoding into an ongoing inventory of complex tropical biodiversity. Molecular Ecology Resources 9(Suppl. 1): 1–26. 10.1111/j.1755-0998.2009.02628.x 21564960

[B26] Kõljalg U Nilsson RH Abarenkov K Tedersoo L Taylor AFS Bahram M Bates ST Bruns TD Bengtsson-Palme J Callaghan TM Douglas B Drenkhan T Eberhardt U Dueñas M Grebenc T Griffith GW Hartmann M Kirk PM Kohout P Larsson E Lindahl BD Lücking R Martín MP Matheny PB Nguyen NH Niskanen T Oja J Peay KG Peintner U Peterson M Põldmaa K Saag L Saar I Schüßler A Scott JA Senés C Smith ME Suija A Taylor DL Telleria MT Weiss M Larsson K-H (2013) Towards a unified paradigm for sequence‐based identification of fungi. Molecular Ecology 22(21): 5271–5277. 10.1111/mec.12481 24112409

[B27] Liu Z He JH Chen XX (2018) The *laevigata*-group of the genus *Dolichogenidea* Mason, 1981 from China, with descriptions of 26 new species. Zootaxa 4436(1): 1–74. 10.11646/zootaxa.4436.1.1 30313169

[B28] Liu Z He JH Chen XX Gupta A Moghaddam MG (2019) The *ultor*-group of the genus *Dolichogenidea* Viereck (Hymenoptera, Braconidae, Microgastrinae) from China with the descriptions of thirty-nine new species. Zootaxa 4710(1): 1–134. 10.11646/zootaxa.4710.1.1 32230513

[B29] Liu Z He JH Chen XX Gupta A (2020) The *ater*-group of the genus *Apanteles* Foerster (Hymenoptera, Braconidae, Microgastrinae) from China with the descriptions of forty-eight new species. Zootaxa 4807(1): 1–205. 10.11646/zootaxa.4807.1.1 33056002

[B30] Lücking R Dal Forno M Moncada B Coca LF Vargas-Mendoza LY Aptroot A Lawrey JD (2017) Turbo-taxonomy to assemble a megadiverse lichen genus: seventy new species of *Cora* (Basidiomycota: Agaricales: Hygrophoraceae), honouring David Leslie Hawksworth’s seventieth birthday. Fungal Diversity 84(1): 139–207. 10.1007/s13225-016-0374-9

[B31] Lücking R Aime MC Robbertse B Miller AN Ariyawansa HA Aoki T Cardinali G Crous PW Druzhinina IS Gieser GM Hawksworth DL Hyde KD Irinyi L Jeewon R Johnston PR Kirk PM Malosso E May TW Meyer W Öpik M Robert V Stadler M Thines M Vu D Yurkov AM Zhang N Schoch CL (2020) Unambiguous identification of fungi: where do we stand and how accurate and precise is fungal DNA barcoding? IMA Fungus 11(1): 1–32. 10.1186/s43008-020-00033-z PMC7353689 32714773

[B32] Marsh P Wild A Whitfield J (2013) The Doryctinae (Braconidae) of Costa Rica: genera and species of the tribe Heterospilini. ZooKeys 347: 1–474. 10.3897/zookeys.347.6002 PMC3822444 24222723

[B33] Meier R Blaimer BB Buenaventura E Hartop E von Rintelen T Srivathsan A Yeo D (2021) A re-analysis of the data in Sharkey et al.’s (2021) minimalist revision reveals that BINs do not deserve names, but BOLD Systems needs a stronger commitment to open science. Cladistics: 1–12. 10.1111/cla.12489 34487362

[B34] Meierotto S Sharkey MJ Janzen DH Hallwachs W Hebert PDN Chapman EG Smith MA (2019) A revolutionary protocol to describe understudied hyperdiverse taxa and overcome the taxonomic impediment. Deutsche Entomologische Zeitschrift 66(2): 119–145. 10.3897/dez.66.34683

[B35] Pinheiro HT Moreau CS Daly M Rocha LA (2019) Will DNA barcoding meet taxonomic needs? Science 365(6456): 873–874. 10.1126/science.aay7174 31467214

[B36] Rakotoarison A Scherz MD Glaw F Koehler J Andreone F Franzen M Glos J Hawlitschek O Jono T Mori A Ndriantsoa SH Raminosoa NRP Riemann JC Rödel MO Rosa GM Vieites DR Crottini A Vences M (2017) Describing the smaller majority: integrative taxonomy reveals twenty-six new species of tiny microhylid frogs (genus *Stumpffia*) from Madagascar. Vertebrate Zoology 67(3): 271–398.

[B37] Ratnasingham S Hebert PD (2013) A DNA-based registry for all animal species: the Barcode Index Number (BIN) system. PLoS ONE 8(7): e66213. 10.1371/journal.pone.0066213 PMC3704603 23861743

[B38] Renner SS (2016) A return to Linnaeus’s focus on diagnosis, not description: the use of DNA characters in the formal naming of species. Systematic Biology 65(6): 1085–1095. 10.1093/sysbio/syw032 27146045

[B39] Riedel A Sagata K Suhardjono YR Tänzler R Balke M (2013a) Integrative taxonomy on the fast track – towards more sustainability in biodiversity research. Frontiers in Zoology 10: e15. 10.1186/1742-9994-10-15 PMC3626550 23537182

[B40] Riedel A Sagata K Surbakti S Tänzler R Balke M (2013b) One hundred and one new species of *Trigonopterus* weevils from New Guinea. ZooKeys 280: 1–150. 10.3897/zookeys.280.3906 PMC3677382 23794832

[B41] Riedel A Tänzler R Balke M Rahmadi C Suhardjono YR (2014) Ninety-eight new species of *Trigonopterus* weevils from Sundaland and the Lesser Sunda Islands. ZooKeys 467: 1–162. 10.3897/zookeys.467.8206 PMC4296478 25610340

[B42] Riedel A Tänzler R (2016) Revision of the Australian species of the weevil genus *Trigonopterus* Fauvel. ZooKeys 556: 97–162. 10.3897/zookeys.556.6126 PMC4740874 26877696

[B43] Riedel A Narakusumo RP (2019) One hundred and three new species of *Trigonopterus* weevils from Sulawesi. ZooKeys 828: 1–153. 10.3897/zookeys.828.32200 PMC6418079 30940991

[B44] Sharkey MJ Meierotto S Chapman E Janzen DH Hallwachs W Dapkey T Solis MA (2018) *Alabagrus* Enderlein (Hymenoptera, Braconidae, Agathidinae) species of Costa Rica, with an emphasis on specimens reared from caterpillars in Area de Conservación Guanacaste. Contributions in Science (Los Angeles County Museum of Natural History) 526: 31–180. 10.5962/p.320146

[B45] Sharkey MJ Janzen DH Hallwachs W Chapman EG Smith MA Dapkey T Brown A Ratnasingham S Naik S Manjunath R Perez K Milton M Hebert P Shaw SR Kittel RN Solis MA Metz MA Goldstein PZ Brown JW Quicke DLJ van Achterberg C Brown BV Burns JM (2021a) Minimalist revision and description of 403 new species in 11 subfamilies of Costa Rican braconid parasitoid wasps, including host records for 219 species. ZooKeys 1013: 1–665. 10.3897/zookeys.1013.55600 34512087 PMC8390796

[B46] Sharkey M Brown B Baker A Mutanen M (2021b) Response to Zamani et al. (2020): The omission of critical data in the pursuit of “revolutionary” methods to accelerate the description of species. ZooKeys 1033: 191–201. 10.3897/zookeys.1033.66186 33958926 PMC8084859

[B47] Srivathsan A Hartop E Puniamoorthy J Lee WT Kutty SN Kurina O Meier R (2019) Rapid, large-scale species discovery in hyperdiverse taxa using 1D MinION sequencing. BMC Biology 17(1): 1–20. 10.1186/s12915-019-0706-9 31783752 PMC6884855

[B48] Summers MM Al-Hakim II Rouse GW (2014) Turbo-taxonomy: 21 new species of Myzostomida (Annelida). Zootaxa 3873(4): 301–344. 10.11646/zootaxa.3873.4.1 25544226

[B49] Taylor JW Jacobson DJ Kroken S Kasuga T Geiser DM Hibbett DS Fisher MC (2000) Phylogenetic species recognition and species concepts in fungi. Fungal Genetics and Biology 31(1): 21–32. 10.1006/fgbi.2000.1228 11118132

[B50] Valerio AA Whitfield JB (2015) Taxonomic review of the genus *Hypomicrogaster* Ashmead (Hymenoptera: Braconidae: Microgastrinae), with descriptions of 40 new species. Zootaxa 3979(1): 1–98. 10.11646/zootaxa.3979.1.1 26249935

[B51] Vences M (2020) The promise of next-generation taxonomy. Megataxa 1(1): 35–38. 10.11646/megataxa.1.1.6

[B52] Yu DSK Van Achterberg C Horstmann K (2016) Taxapad 2016, Ichneumonoidea 2015. Database, Ottawa.

[B53] Zamani A Vahtera V Sääksjärvi IE Scherz MD (2020) The omission of critical data in the pursuit of ‘revolutionary’ methods to accelerate the description of species. Systematic Entomology 46: 1–4. 10.1111/syen.12444

