# Supplementary material for: ﻿Turbo taxonomy approaches: lessons from the past and recommendations for the future based on the experience with Braconidae (Hymenoptera) parasitoid wasps
Source: Zookeys. 2022 Feb 25;1087:199–220. doi: 10.3897/zookeys.1087.76720 (PMC8897373; doi:10.3897/zookeys.1087.76720)
Supplement: Supplementary material 1 — Comments from the reviewers [file zookeys-1087-199-s001.docx]

**Supplementary File**

**Comments from the reviewers of the manuscript, ordered as they were submitted. All reviewers agreed to be named in this supplementary file.**

1. **Brian V. Brown**

**Taxonomic Expertise: Diptera**

**Curator, Entomology Section**

**Natural History Museum of Los Angeles County, USA**

This is a well-written, reasonable paper. I disagree with the author on some points, however, and hope he will consider revising the paper in light of these arguments.

1. The argument is made that sequencing costs are too expensive. The example is given that, in order to get a single specimen barcoded, you need to pay for a full 96-well plate at BOLD. This is incorrect; researchers wanting a single sequence can extract and pcr a gene region and submit it for Sanger sequencing for ~ $10 at many sequencing companies throughout North America.

2. The argument is made that ~ 100 species per year is reasonable output for turbo taxonomy. In my group, the family Phoridae, we have just estimated that there are some 700,000 world species. How will we deal with this type of diversity?

3. One factual error is that Hartop and Brown have not gone on to use their version of turbo taxonomy, other than for one species. This is incorrect, we have described (a rather unimpressive) 50 or so species using this model.

4. The statement is made that “all body areas” should be imaged. What is meant by “all”, and what detail is needed? Surely this is more subjective than what the author declares.

5. There is a section on “hidden” costs of DNA taxonomy that are not disclosed. Sharkey, for instance, has much of his databasing costs “subsidized” by working from BOLD material. For others, however, there might be costs that are not stated. My comment on this is that big taxonomy like this is no more possible without support (money) that is other big science. The idea of taxonomists working in poverty, with no grant support, is just not reasonable. We do not expect other scientists to work in this way, so why should taxonomists? I would say that if you wan to work on one of these hyperdiverse groups, you should get some funding, or stay out of them.

6. The biggest shortcoming of this paper, and those of others critical of “fast” taxonomy, is the author’s apparent refusal to envisage high volume taxonomy. I suggest that for these extremely species-rich groups, we abandon the idea that anyone can (or should) be able to identify a couple of random specimens that they have on hand using morphology. That is not possible now, and certainly will not be possible at any time in the foreseeable future. Why should we waste our time writing keys (even to groups) that nobody will ever use? On the other hand, metabarcoders and others doing high-volume biodiversity work could use our DNA-based descriptions to start to understand whole faunas. Taxonomy in these groups should be a high-volume process in my opinion, and move into the modern world. A single taxonomist sorting through a unit tray of braconids will not be able to identify them to species, but as I said, they cannot do that now.

1. **Stefan Schmidt**

**Taxonomic Expertise: Hymenoptera**

**Head of Hymenoptera Section**

**Bavarian State Collection of Zoology, Munich, Germany**

The author discusses recent attempts to accelerate the process of taxonomic descriptions of new species, referred to as "turbo taxonomy". As one of the most productive taxonomists with a remarkable output record, the author is certainly in a position to articulate his opinion about recent attempts to put "turbo taxonomy" into practice. Most importantly, he elaborates on an aspect that has, to my knowledge, never been dealt with in any previous publication on this topic, but that is agreeably most pertinent when discussing revisionary studies that claim to have accelerated the taxonomic procedure of describing new species, often employing a combination of morphological and molecular tools. These studies usually do not include the time needed for the often laborious groundwork and preparatory work that has been done before the turbo part started, like collecting, rearing, mounting, labelling etc. The author then argues that 1) turbo taxonomy revisions are less turbo than they claim to be, and 2) they could have avoided major flaws that have been heavily criticized by investing a little bit more effort into improving the quality (and hence usability) of species descriptions. The attempt to reconcile the different approaches and to rationalise the heated discussion sets this Forum Paper apart from other commentary papers, that, in my opinion, often pour the child out with the bath.

Perhaps it is because of my previous experience with taxonomy and DNA barcoding that I regard the recipes provided by the author proposes as very sensible, because he tries to reconcile the different methods by combining the best parts of each of the different approaches in a constructive and efficient way (taking all factors into account!). It helps that the author provides rather detailed information about the time requirements of each step, something that is usually lacking from similar contributions. It is appreciated that the fast-track taxonomy proposal tries to reconcile the different approaches, traditional and advanced, targeted at the “golden mean”, and I admit to being on the same page with the author in this respect.

Something I would have liked to see are some comments on to what extent the time needed for completing cited extreme turbo-taxonomy examples are miscalculated not only because they omit the “hidden” previous work by others but also because they impose more work for future taxonomists (called “description impediment” by Meier et al. 2021). Saving time today by omitting important aspects in the workflow (like proper documentation of samples, specimen labelling, insufficient descriptions, poor quality images, etc.) often need more time to be sorted out in the future (apart from being additional sources or errors), time that could have been better spent by doing things properly in the first place.

In summary, this Forum Paper is a welcome addition to the somewhat overheated discussion on how to proceed with current taxonomy. I added few comments but since it is primarily an opinion paper, I will leave it to the author to decide whether include them or not, and recommend the ms for publication “as is”.

1. **Marko Mutanen**

**Taxonomic Expertise: Lepidoptera, Hymenoptera**

**Senior Curator**

**Ecology and Genetics Research Unit, University of Oulu, Oulu, Finland**

José Fernandez-Triana has written a very nice opinion piece about “turbo taxonomy”, with many useful recommendations for future directions. This topic is presently hotly debated, and I find this paper a very good and constructive addition to this debate. I especially like that it seeks for compromises and better practices instead of blaming any party’s aspects.

From the acknowledgements, I understand that this paper has already been reviewed by two reviewers. The paper could be published as it presently stands, but I provide some further insights that the author could consider if he wished so. However, I would not necessitate my ideas being considered because, as mentioned, the logic presently is already mostly very solid.

Fernandez-Triana focusses largely on Braconidae and takes examples from there, but as the title suggests, the article is planned to provide a more widely applicable model. I find the proposed approach as an attempt to bridge between those defending traditional approaches and those defending the adoption of wider use of DNA barcoding in taxonomy. Hence, the solution is in practice to augment the turbo taxonomy with more comprehensive consideration morphology, ecology, biology, and other non-barcode sources of information. Fernandez-Triana aims to demonstrate that it would be efficient and feasible to combine the benefits of these approaches. He also strongly speaks for the prior taxonomy being considered better than in some previous works. Very fair demands.

Related to this, there are three aspects that to my opinion have not been considered very well. First, the number and proportion of priorly described species vary a lot from area to area and group to group. When we then get an overview of barcode OTUs based on turbo taxonomic approach, it sometimes does not require a lot to consider the previously described species as they are few. This seems to be largely the case in Costa Rica, for instance. But in Europe, this problem is often a huge one. I know of numerous cases, and I am facing many myself, where the description of an evidently new species has been blocked only by the “historical backlog”, i.e., the availability of a plenty of prior names and synonyms that are difficult to interpret due to their poor condition, poor availability, lack of genetic data, etc. That all the previous species, and particularly synonyms, should be investigated makes taxonomic work extremely challenging and time-consuming, and in practice prevents the progress. Taxonomists should not be historians. To my opinion, this is a major component of the taxonomic impediment. We should find more efficient ways to deal with the old names (or rather, to ignore them). A possible solution would be to conduct massive sequencing of all type specimens as efficient methods to recover DNA from old specimens exist, but sometimes even this would not help.

Second, considering morphology of already described species may sound a feasible goal, but it is important to note that this part of the work is cumulating, finally making it extremely complicated and time-consuming. It has been estimated that there are 1.8 million species of gall midges. Who would compare morphology of, e.g., 300,000 species when describing a new one? Who would make a key for them all? Who would use such a mega-key? Morphology and its being poorly quantifiable cannot remain a critical part of species descriptions forever. It is exactly because of that reason that perhaps 90% of species remain undescribed. We have started from the easiest ones, and the difficult ones remain.

Third, while Braconids may provide useful clues for morphology, this is reduced in many other groups that also deserve taxonomy to be worked. Think of perhaps 200,000 species of Nematode worms awaiting description, all more or less alike and with few structures for morphological comparison. Why should we even consider their morphology? I believe that for 80% of the undescribed fraction of species, morphology will never provide an efficient tool to tell species apart. Morphological keys inevitably become complex when the number of species grow high, rendering them quite useless.

It is important to note that all taxonomically relevant information is encoded in DNA and that this information is fully quantifiable. Computers can efficiently, in a few seconds, compare DNA across millions of specimens and species. This is not at all true for morphology, and even less so for ecology, food, etc. I fully acknowledge that DNA barcodes are not enough to provide a fully accurate picture of species diversity. However, instead of spending time (and money as time is valuable as well) on morphology, why not spend that time on other genetic markers? This would provide independent (if from nuclear DNA) evidence for defining the species boundaries. I agree with Fernandez-Triana that DNA methods are not accessible to everyone and require funds, but these technologies are developing in an unprecedented pace. Illustratively, while Fernandez-Triana uses the Sanger-based cost estimate of barcoding (1250 CAD per 95 specimens), I am already now doing barcoding at a massive scale with a price of approximately one fourth of this using the SEQUEL platform that CCDB also offers for customers. My student recently analyzed more than 9200 specimens of gall midges using this approach. This analysis yielded in excess of 1400 BINs in an area with ~ 380 from previously known species. This all was conducted in a few months. Accumulating an equal amount of taxonomically useful data by traditional means would probably require an entire lifetime. And that all would become, all costs considered, much more expensive. While our gall midge species remain undescribed, I am sure that gathering any comparable data sorted out to this level also could not have been done as efficiently by any other means.

I find that overcoming the taxonomic impediment would require much more radical approaches. It would require the rules to be accommodated to provide efficient solutions to ignore the dusty history, old names, poorly interpretable types and so on. It would require adoption of high-throughput genetic/genomic tools. It would require a shift from integrative approach to DNA taxonomy as recently proposed by Eberle et al. and Dietz et al. (Eberle, J., Ahrens, D., Mayer, C., Niehuis, O. & Misof, B. 2020. A plea for standardized nuclear markers in metazoan DNA taxonomy. Trends Ecol. Evol. 35: 336–345.; Dietz, L., Eberle, J., Mayer, C., Kukowka, S., Bohacz, C. et al. 2021. Standardized nuclear markers advance metazoan taxonomy. BioRxiv 2021.05.07.443120). I understand that these would be radical proposals and realize that few taxonomists would agree with me with these ideas. But I think that it would be important to better recognize what a hopeless enterprise it is to continue basing taxonomy on morphology or integrative approaches. It simply will not allow us to finish this work, at least in a reasonable time, but more likely never. I would not bet the Linnean system to resist forever. Once we can read biodiversity by “barcodes” (perhaps augmented by additional markers) better than we can do by morphology, the shift will happen automatically. This shift has actually already taken place with microbes. If sequencing technology continues to develop as it has done during the last few decades, accumulating huge amounts of genetic data from millions of specimens cheaply becomes a realistic scenario. Who would then continue manually tabulating morphological features or measurements to an Excel file with the same effort and money as one could read DNA from large numbers of individuals and this way accumulate data much more efficiently directly from the genome? Data that is quantifiable and allows assessing e.g., degrees of gene flow.

The description of requirements of barcoding work overestimates the required time and resources because this follows strict standards for iBOL for building a reference library. When doing sequencing only for taxonomic research, there is no need to photograph all specimens. In the mentioned gall-midge example by us, for example, we plan to photograph only representatives of each cluster in the connection of taxonomic work (though fast mass photography will also be conducted). Some species are represented by hundreds of individuals in our data. We are not going to photograph them all, not least with high precision. This kind of research that can be conducted outside the strict iBOL framework would enable analyses of tens of thousands of specimens with minimal effort and focusing working on details only when this is really necessary.

Fernandez-Triana also discusses that turbo taxonomy requires a remarkable work required prior to the taxonomic study. This is true, but this work is often nevertheless m in connection with other studies, projects, or by amateur collectors. Additionally, collecting huge numbers of specimens is not a major issue in many groups, parasitoids included. Again, our gall midge study demonstrated this well. With Malaise traps, we could collect and pick thousands of specimens in a few months. Sequencing thousands of those specimens as full specimens (non-destructively e.g., with the SEQUEL approach) does not require a huge amount of time or very much funding. Research institutes are typically supported by personnel who can assist in such routine work. I have myself benefitted from students as well, cheap and efficient.

With these ideas, I certainly do not mean that Fernandez-Triana’s proposal would not be a great one. It is a proposal that I certainly recommend and find it a big step forward. The ideas I discuss are largely my own ones and I would not require Fernandez-Triana to consider them. But I am of course happy if he finds any of my ideas useful and worth considering.

1. **Istvan Miko**

**Taxonomic Expertise: Hymenoptera**

**Collections Manager**

**University of New Hampshire, Durham, USA**

I am really sorry for not being able to turn in my review in time. I read the MS multiple times, but struggled to write up a proper review or give any recommendations. I agree that the MS is great and perhaps it could be published as it is, but have the feeling that it is, at some points, too lengthy and also too personal and sections like the “Talking the talk and walking the walk” and “Speed, practicality, affordability, democratization of taxonomy and Star Trek'' just distract the reader and draw the attention from the two really useful sections (comparative review of turbo taxonomy papers in Braconidae and the proposed workflow). I think the second makes this paper especially valuable, as it, unlike other already published workflows for turbo taxonomy, has already been proven successful as José has applied it on multiple occasions. It is just difficult to understand why sections like the one from the Introduction starting with “I do not claim to have better or newer insights than others, and I certainly do not pretend to have any definitive answers…” (including José's comment on reviewing the Sharkey et al. paper) are necessary, in fact they are just disrupting the reader. I do not think José should apologize for writing down his opinion, in fact, that turbo taxonomy community (especially the ichneumonoid one) should apologize about why they did not pay more attention to his awesome work.

But it is, of course, your decision, the paper is well written and would be very important to publish and I would definitely cite it in my next revision (I have very similar issues when working with ceraphronids from BOLD).
